# How discriminating are discriminative instruments?

**DOI:** 10.1186/1477-7525-6-36

**Published:** 2008-05-27

**Authors:** Matthew Hankins

**Affiliations:** 1 King's College London, Department of Psychology (at Guy's), Institute of Psychiatry, London, UK; 2 Department of Primary Care & Public Health, Brighton & Sussex Medical School, Brighton, UK; 3 Brighton & Sussex University Hospitals NHS Trust, Royal Sussex County Hospital, Brighton, UK

## Abstract

The McMaster framework introduced by Kirshner & Guyatt is the dominant paradigm for the development of measures of health status and health-related quality of life (HRQL). The framework defines the functions of such instruments as evaluative, predictive or discriminative. Evaluative instruments are required to be sensitive to change (responsiveness), but there is no corresponding index of the degree to which discriminative instruments are sensitive to cross-sectional differences.

This paper argues that indices of validity and reliability are not sufficient to demonstrate that a discriminative instrument performs its function of discriminating between individuals, and that the McMaster framework would be augmented by the addition of a separate index of discrimination. The coefficient proposed by Ferguson (Delta) is easily adapted to HRQL instruments and is a direct, non-parametric index of the degree to which an instrument distinguishes between individuals. While Delta should prove useful in the development and evaluation of discriminative instruments, further research is required to elucidate the relationship between the measurement properties of discrimination, reliability and responsiveness.

## Background

The McMaster framework [1] defines the functions of health status instruments as evaluative, predictive, or discriminative. *Evaluative *instruments measure longitudinal change, typically the effects of treatment. *Predictive *instruments are used classify individuals against an external criterion and are intended for diagnostic, prognostic or screening purposes. *Discriminative *instruments are used to quantify differences between individuals when no external criterion exists, typically in cross-sectional studies. These functional definitions have been widely adopted as the methodological basis for the measurement of health-related quality of life (HRQL) [2].

The validity of a HRQL instrument depends primarily on the instrument measuring the correct aspect of HRQL [3]. This is usually demonstrated by appropriate correlations with other measures [4]. Beyond this, the framework specifies that the necessary measurement properties for validity depend on the intended function of the instrument [1].

Evaluative instruments are required to give consistent measurements (reliability) and be sensitive to change (responsiveness) [3]. Reliability may be estimated by a variety of methods [4], but for longitudinal consistency the usual method is a test-retest correlation or intra-class correlation (ICC) [3]. Responsiveness is usually indexed by a mean difference adjusted for variance (for example, Cohen's *d *or the standardised response mean [5]).

Discriminative instruments, in contrast, are required only to be reliable. Since they are used primarily in cross-sectional samples, reliability is commonly estimated using cross-sectional data, typically Cronbach's Alpha (a two-way mixed effects ICC [6]). If longitudinal data are available, another estimate may be derived from the test-retest correlation or ICC [6]. As discriminative instruments are not used to measure change, they are not required to be responsive [3].

The reliability of an evaluative instrument does not tell us how sensitive it is to longitudinal differences [3]. Similarly, it may be argued that the reliability of a discriminative instrument fails to tell us how sensitive it is to cross-sectional differences. For example, an instrument might consistently fail to discriminate between people (reliable but not discriminating), or discriminate well, but inconsistently (discriminating but not reliable). This paper proposes therefore that the McMaster framework would be augmented by an additional index of discrimination. The implications of such an index for the development of discriminative instruments will be discussed, with examples.

### Indices of discrimination

The sort of discrimination required of discriminative instruments is known in the classical psychometric literature as *test discrimination *[7]. This is the ability of a psychometric test to be able to distinguish between individuals without reference to an external criterion. In contrast, *discriminant validity *requires an external criterion, which is more consistent with the framework's definition of a predictive instrument [4], and *item discrimination *refers to the difficulty of each item of the test [8,9].

The earliest attempts to describe test discrimination were based on 'cumulative' scales such as Guttman scales [10]. Walker [11] and Loevinger [12,13] developed coefficients to describe the degree to which the scale approached the psychometric ideal that a score of *n *indicated that the least difficult *n *items had been answered correctly, and no others. Taking an atheoretical approach, Thurlow [14,15] and Ferguson [7] both recognised that for a given sample size there would be a maximum possible number of differences that might be observed. This could be compared with the number of differences actually observed and expressed as a ratio: the coefficient of discrimination. Thurlow seems to have been the first to recognise the distinction between discrimination and reliability, but despite presenting the coefficient (and modifications of it) earlier and treating the issue of discrimination in considerably more depth, it is commonly referred to as "Ferguson's Delta" [4].

Ferguson's Delta is the ratio of the observed between-persons differences to the maximum number possible. If no differences are observed, then Delta = 0.0; if all possible between-person discriminations are made, then Delta = 1.0. Delta is not restricted to Guttman scales and is non-parametric, being based solely on the ordinal properties of the data. It has one limitation that has restricted its use with a wider range of questionnaire measures: the scale must comprise dichotomous (binary) items. Fortunately, this limitation is easily overcome [16] making Delta more widely applicable to HRQL instruments.

### Calculation of Delta

Ferguson's original formula (simplified by Guilford [17]) is appropriate for scales with dichotomous items:

(1) $$\delta =\frac{\left(k+1)(n^{2}-\sum_{i}f_{i}^{2}\right)}{kn^{2}}$$

where *k *is the number of items, *n *is the sample size and *f *is the frequency of each score *i *(with *i *ranging from 0 to *k*).

For scales with more than two response options (such as Likert scales), the modified formula should be used [16]:

(2) $$\delta =\frac{\left(1+k(m-1))(n^{2}-\sum_{i}f_{i}^{2}\right)}{n^{2}k(m-1)}$$

where *m *is the number of item responses and all other terms remain the same. For a typical Likert scale, *m *= 5.

The calculation of Delta is relatively straightforward: an Excel spreadsheet is available, as well as program code in R (with bootstrapped 95% confidence limits) and Stata [16].

### Examples of discrimination analysis

#### Example 1: worked calculation of Delta

To illustrate the calculation of Delta, consider two equally valid single-item Likert instruments, Scale A and Scale B, given to 10 people known to differ in HRQL. Responses to Scale A are 1,2,2,3,3,3,3,4,4,5 and responses to Scale B are 1,1,1,1,1,1,1,1,3,5. The scales agree substantially (ICC = 0.83). Since the scale is not dichotomous, formula (2) is required, with values *k *= 1, *m *= 5 and *n *= 10.

Table 1 gives the frequency tables for Scales A and B. It should be obvious that, despite their high concordance, Scale B is the less discriminating of the two, since eight people are not discriminated from each other (all scoring 1). From the formula, Scale A Delta = (1+4) * (100-26)/400 = 0.925, and for Scale B Delta = (1+4)*(100-66)/400 = 0.425. Hence, Scale A makes 92.5% of all possible discriminations, while Scale B makes only 42.5% of all possible discriminations: Scale A is almost twice as discriminating as Scale B. Ferguson [7] suggested that a normal distribution would be expected to have discrimination of Delta > 0.90, with lower discrimination expected for leptokurtic and skewed distributions (since leptokurtic distributions fail to discriminate around the mean, while skewed distributions fail to discriminate at one end of the distribution). On this basis, Scale A shows good discrimination while Scale B shows poor discrimination.

**Table 1:** Hypothetical data for two single-item Likert instruments (n = 10)

Scale A			Scale B		
Score *i*	Frequency *f*_*i*_	$f_{i}^{2}$	Score *i*	Frequency *f*_*i*_	$f_{i}^{2}$

1	1	1	1	8	64
2	2	4	2	0	0
3	4	16	3	1	1
4	2	4	4	0	0
5	1	1	5	1	1

Sum		26			66

#### Example 2: reliability and discrimination of self-report instruments

For further examples, data were obtained for the 2004 cohort of the Health Survey for England (HSE [18]: usage ID 21697). Details of sampling and methodology are publicly available and the data are used here for demonstration only. Since the HSE samples by household, the records were filtered to produce a dataset of the 4000 'reference' adults in the household to ensure that the data were independent.

The HSE includes a number of self report instruments of interest to HRQL researchers: Table 2 shows those selected for this analysis, describing the number of items (*k*), number of item response options (*m*) and the number of people completing (*n*). For the purposes of demonstration, a range of instruments was chosen: a single-item Likert-type scale (self-reported health), a multi-item scale with dichotomous response options (GHQ-12 [19]), a multi-item scale with polytomous response options (Perceived Social Support [20]) and a non-summative multi-item scale with polytomous response options (EuroQoL [21]).

**Table 2:** Selected self-report instruments from the Health Survey for England (2004 cohort, N = 4000)

Scale	Number of Items (*k*)	Number of response options (*m*)	Number completing (*n*)	Alpha	Delta
Self-reported health	1	5	3997	-	0.84
GHQ-12	12	2	3705	0.88	0.63
Perceived Social Support	7	3	3730	0.88	0.64
EuroQol	5	3	3679	0.77	0.71

Since the data were cross-sectional, reliability was estimated using Cronbach's Alpha. Alpha and Delta values are also presented in Table 2. For the single item self-reported health instrument it was not possible to compute Alpha; the value for Delta, however, suggested that the instrument was discriminating, with 84% of possible discriminations being made. The reliability of the GHQ-12 (scored dichotomously) was acceptable (Alpha = 0.88), but discrimination was poor (Delta = 0.63) with less than two thirds of possible discriminations being made. A similar result was found for the Perceived Social Support instrument: acceptable reliability (Alpha = 0.88) but poor discrimination (Delta = 0.64). The EuroQol instrument demonstrates the versatility of Delta as an index of discrimination. The EuroQol assess quality of life in five dimensions using five items, each with three response options coded 1 to 3. Responses to each item are not summed in the usual manner but are used to describe a unique 'health state': for example 11111 is a different health state to 11221. Since there are five items each with three responses, there are 243 possible health states, ranging from 11111 (best) to 33333 (worst). Although Alpha may be calculated to demonstrate the consistency of responses to items, it describes the reliability of the summed score, not the classification of health state. Delta, however, may still be used since different health states may be discriminated from each other. The EuroQol showed acceptable reliability for the summed score (Alpha = 0.77), but less than optimal discrimination between health states (Delta = 0.71).

#### Example 3: instrument development

The tabulated data (Table 3) show item scores, reliability and discrimination for an eight item dichotomous test of numeracy. Also shown are the reliability and discrimination of the instrument when each item is removed. The data were obtained as part of a study of health-related numeracy [22] unrelated to this paper and are used for illustration only. Once again the data are cross-sectional and Alpha is used as an estimate of reliability. Dichotomously-scored instruments are not common in HRQL measurement, but the principles illustrated here apply equally to Likert-type scoring.

**Table 3:** Reliability and discrimination of the eight item Lipkus numeracy scale (N = 140)

Item	Alpha if item	Delta if item	Delta for item	Short form A	Short form B	Short form C
	deleted	deleted				
Q1	0.74	0.88	0.97		+	+
Q2	0.77	0.90	0.72		+	
Q3	0.71	0.92	0.49	+		+
Q4	0.70	0.91	0.71	+		+
Q5	0.71	0.92	0.40	+		+
Q6	0.71	0.92	0.43	+		
Q7	0.74	0.91	0.72		+	
Q8	0.76	0.85	0.99		+	
Scale Alpha	**0.76**			**0.85**	**0.51**	**0.72**
Scale Delta	**0.92**			**0.56**	**0.95**	**0.82**

The problem considered is that of item reduction: a researcher is required to shorten the instrument from eight items to four to alleviate the burden on respondents. Three alternative short forms of the instrument (A, B and C) are presented in Table 3 with their reliability and discrimination coefficients.

Short form A is the instrument that results from retaining items solely for their impact on reliability (items Q3 to Q6). This has the effect of increasing reliability (Alpha = 0.85), but drastically decreasing discrimination (Delta = 0.56). The selection of items solely on the basis that they are highly consistent with each other results in a discriminative instrument that makes only 56% of the possible number of discriminations.

In contrast, short form B is the instrument that results from retaining items solely for their impact on discrimination (items Q1, Q2, Q7 and Q8). This maintains the discrimination of the instrument (Delta = 0.95) but decreases reliability to an unacceptable level (Alpha = 0.51).

A compromise is required: short form C comprises items selected on the basis of their impact on both reliability and discrimination. Note that this entails a slight loss of both reliability (Alpha = 0.72) and discrimination (Delta = 0.82). Whether or not these indices are sufficient for a specific research project is, of course, a matter for the judgement of the researcher. However, the point should be clear that neither A nor B is likely to be a valid instrument in terms of both reliability and discrimination.

## Conclusion

It seems that the McMaster framework would be augmented by considering the discrimination of discriminative instruments. The results presented here demonstrate that reliable measures may fail to discriminate adequately, and developing measures solely to maximise internal reliability may be counterproductive.

That this aspect of the framework has been neglected may be due to the emphasis in HRQL research on measuring change; hence the focus has been on evaluative instruments, their responsiveness, and refining indices of responsiveness. In other words, if discriminative instruments are rarely developed, then it should not be surprising if little attention has been given to indices of discrimination. The relationship between reliability, validity, responsiveness and discrimination is largely unexplored, particularly for longitudinal measurements. Further research is required into the measurement properties of existing HRQL instruments and the development of new ones. It is hoped that the outline and examples given here will help researchers achieve this aim.

## Abbreviations

GHQ-12: General Health Questionnaire (12-item version); HRQL: Health-related quality of life; HSE: Health Survey for England; ICC: Intraclass correlation coefficient.

## Competing interests

The author declares that he has no competing interests.

## Authors' contributions

MH was the sole author.
